# Latency shortening with enhanced sparseness and responsiveness in V1 during active visual sensing

**DOI:** 10.1038/s41598-022-09405-4

**Published:** 2022-04-11

**Authors:** Junji Ito, Cristian Joana, Yukako Yamane, Ichiro Fujita, Hiroshi Tamura, Pedro E. Maldonado, Sonja Grün

**Affiliations:** 1 Institute of Neuroscience and Medicine (INM-6, INM-10) and Institute for Advanced Simulation (IAS-6) and JARA BRAIN Institute I, Jülich Research Centre, Wilhelm-Johnen Str., 52425 Jülich, Germany; 2 Institut of Mathematics and Physics (IRMP), University of Louvain, Louvain-la-Neuve, Belgium; 3 Graduate School of Frontier Biosciences, Osaka University, Osaka, 565-0871 Japan; 4 Center for Information and Neural Networks, Osaka University and National Institute of Information and Communications Technology, Osaka, 565-0871 Japan; 5 Okinawa Institute of Science and Technology Graduate University, Okinawa, 904-0495 Japan; 6 Department of Neuroscience and Biomedical Neuroscience Institute, Faculty of Medicine, Universidad de Chile, Santiago, Chile; 7 Theoretical Systems Neurobiology, RWTH Aachen University, 52056 Aachen, Germany

**Keywords:** Neuroscience, Visual system, Striate cortex

## Abstract

In natural vision, neuronal responses to visual stimuli occur due to self-initiated eye movements. Here, we compare single-unit activity in the primary visual cortex (V1) of non-human primates to flashed natural scenes (passive vision condition) to when they freely explore the images by self-initiated eye movements (active vision condition). Active vision enhances the number of neurons responding, and the response latencies become shorter and less variable across neurons. The increased responsiveness and shortened latency during active vision were not explained by increased visual contrast. While the neuronal activities in all layers of V1 show enhanced responsiveness and shortened latency, a significant increase in lifetime sparseness during active vision is observed only in the supragranular layer. These findings demonstrate that the neuronal responses become more distinct in active vision than passive vision, interpreted as consequences of top-down predictive mechanisms.

## Introduction

Ever since the pioneering work of David Hubel and Torsten Wiesel in the primary visual cortex of the cat^1,2^, the central paradigm for examining the neuronal activity of visual cortices has been the use of simple stimuli such as a bar or gratings, which are typically flashed or moved across the receptive field of the neurons of interest. This successful paradigm has produced a vast amount of knowledge about how individual neurons respond to this type of stimuli, and with these response properties, many models of visual perception have been constructed^3–7^.

However, natural vision differs substantially from situations studied with the classical paradigm. For example, stimuli outside the classical receptive field extensively modulate responses to stimuli within the receptive fields^8–13^. These considerations have led to a series of studies that examined the neuronal activity of visual cortices in more ecological situations using natural images^14–18^. In addition, during natural vision, visual inputs are acquired actively by self-initiated saccadic eye movements. Previous studies have shown that response properties of neurons in the primary visual cortex (V1) when stimulated by images acquired by self-initiated eye movements differ from those in response to flashed images^19,20^.

We conjectured that eye movements occur within a closed-loop process^21^, where the consequences of motor action directly and/or indirectly affect the activity of the sensory system. Thus, neurons in the visual system should change their neural activity due to both the visual input and sensory-motor mechanisms, the latter of which would be absent in the classical 'passive' paradigm. Therefore we conducted neuronal recordings from V1 of macaque monkeys trained to perform a free viewing task, and compared single-unit responses from V1 during actively viewing natural images to their responses in a passive condition, i.e., to the onset of flashed images while the monkeys kept fixation. We evaluated different aspects of the single-unit activity and found that the neurons' responsiveness, sparseness, and response timing significantly changed from passive to active vision. This highlights the need to further compare results from classical experimental paradigms with more naturalistic settings to understand brain mechanisms.

## Results

In this study, we aim to compare passive and active viewing conditions. Thus, we examine the neuronal responses in two conditions a) to the onset of image presentation (passive), which corresponds to a flashed stimulus, and b) to the onsets of eye fixations after self-initiated eye movements during freely viewing the same image (active). The experimental trials were as follows: first, the monkey had to fixate a fixation spot at the center of a blank screen, during which a naturalistic image was presented. We consider this initial visual response situation as passive vision. Subsequently, the monkeys freely viewed the same image for 3 or 5 s (depending on the monkeys), and every visual fixation following a saccade was considered an instance of active vision. Thus, we evaluate the neuronal spiking responses aligned to the image onset (img-on) in the former case and aligned to the onset of the eye fixations (fix-on) in the latter case.

**Figure 1:**
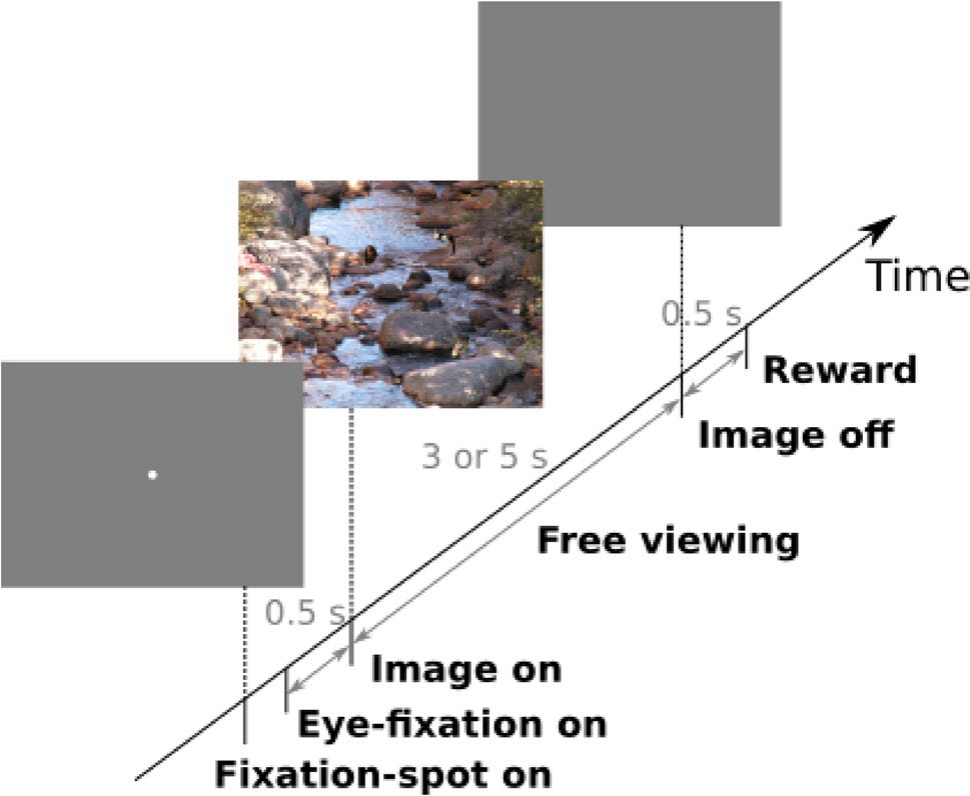
Free-viewing task protocol illustrated as the timeline of a trial. Monkeys are allowed to explore the presented natural scene image (with 5 object images artificially embedded) with voluntary eye movements during the 3 or 5-s free-viewing period. See “Methods” for more details.

**Figure 2:**
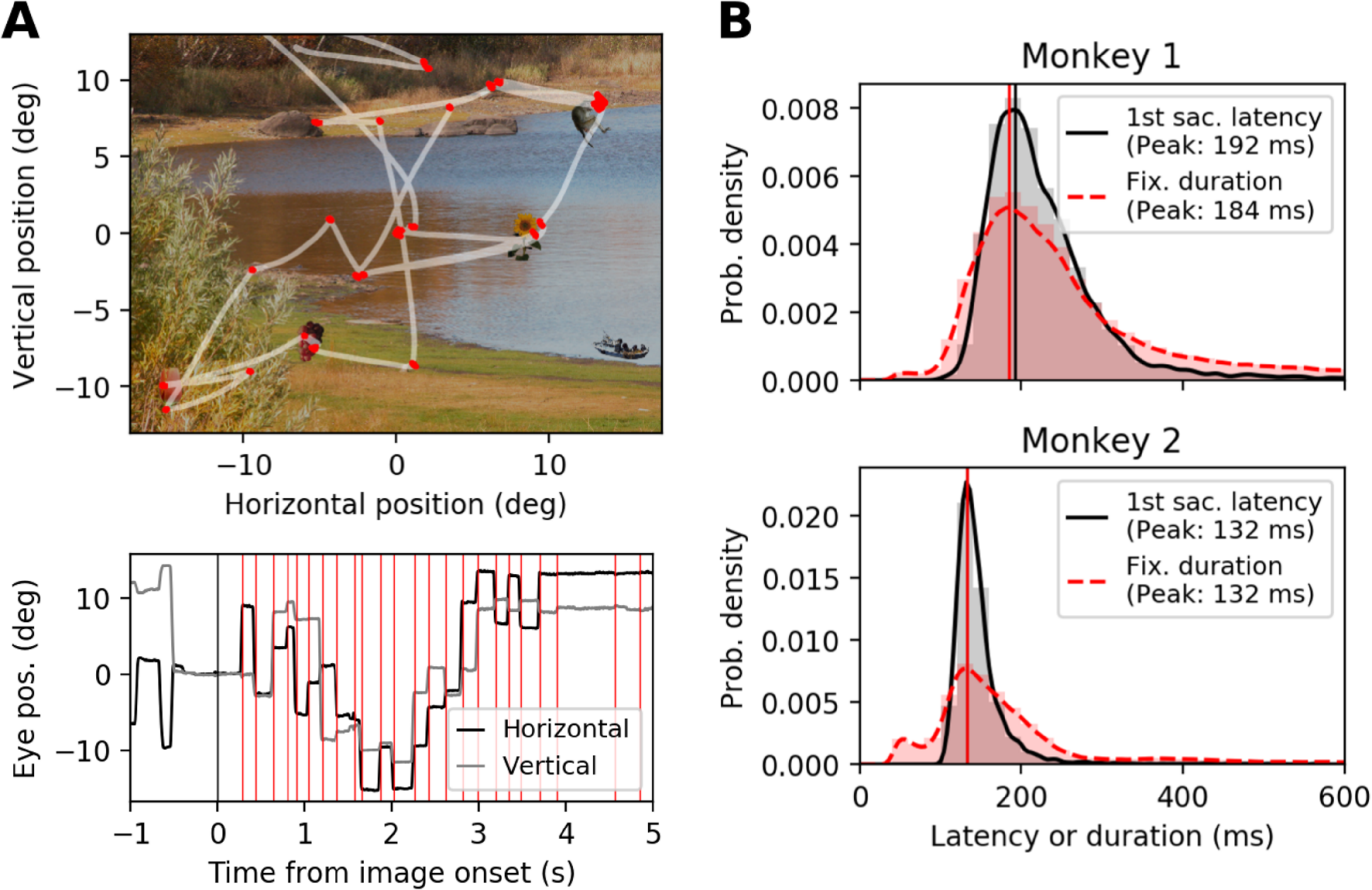
Eye movement characteristics in passive and active viewing conditions. (**A**) Eye movements performed by monkey 2 in an example trial of the free-viewing task. (Top) eye movement trajectory (white) overlaid on the stimulus image used in the trial. Red dots indicate the locations of eye fixations during free-viewing. (Bottom) horizontal (black) and vertical (gray) eye position during the free-viewing shown in the top panel, plotted as a function of time. Black and red vertical lines indicate img-on and fix-on, respectively. (**B**) (Top) distribution of the latencies from img-on to the onset of the first saccade (black), and distribution of the duration of fixations performed during free-viewing (red) for monkey 1. The vertical lines indicate the peak of the respective distributions. (Bottom) Same as the top panel, but for monkey 2.

Two macaque monkeys (monkeys 1 and 2) performed such a free-viewing task (Fig. 1) while their eye movements and neuronal activity in V1 were recorded (see “Methods” for details). We collected these data for 2609 trials over 79 recording sessions from monkey 1 and 3126 trials over 25 recording sessions from monkey 2. As reported previously^15^, both monkeys spontaneously explored the natural images with self-initiated saccades, fixating on several regions of the images, including the objects embedded in the stimulus images (Fig. 2A). Across the sessions, monkeys 1 and 2 made 13.8 ± 3.65 and 17.8 ± 5.68 fixations per trial (mean ± std), respectively.

## Eye movements

To evaluate potential differences in the eye fixation behavior between the two conditions, we compare the distribution of the latencies from img-on to the onset of the first saccade (Fig. 2B, black solid curves) to the distribution of the latencies from fix-on to the onset of the following saccade (Fig. 2B, red dashed curves). The peak positions of the two distributions show that the latencies from img-on to saccade and from fix-on to saccade are very similar within each monkey, but are somewhat different between the two (132 ms for monkey 1 and about 190 ms for monkey 2). In both passive and active conditions, the visual input needs to be processed to plan and perform the next saccade. This corresponds to the first saccade on the image for the passive condition, and in the active condition, this corresponds to one of the following saccades. The above result indicates that the time it takes for such processing is similar between the passive and active conditions.

Besides the peak positions, the latency distributions are narrower for the passive condition than for the active condition (interquartile range: 79.3 ms (passive) vs. 150 ms (active) for monkey 1; 26.5 ms (passive) vs. 90.6 ms (active) for monkey 2). This implies that the processing time after img-on is more consistent across trials than after fix-on across fixations. The wider fixation duration distribution during free-viewing could be related to the fact that during natural vision, two different processing modes are present: during the local mode, i.e., exploration within an object, the saccades are much shorter than during the global mode, when saccades are relatively large and go from one object to another^15^.

### Responsiveness

**Figure 3:**
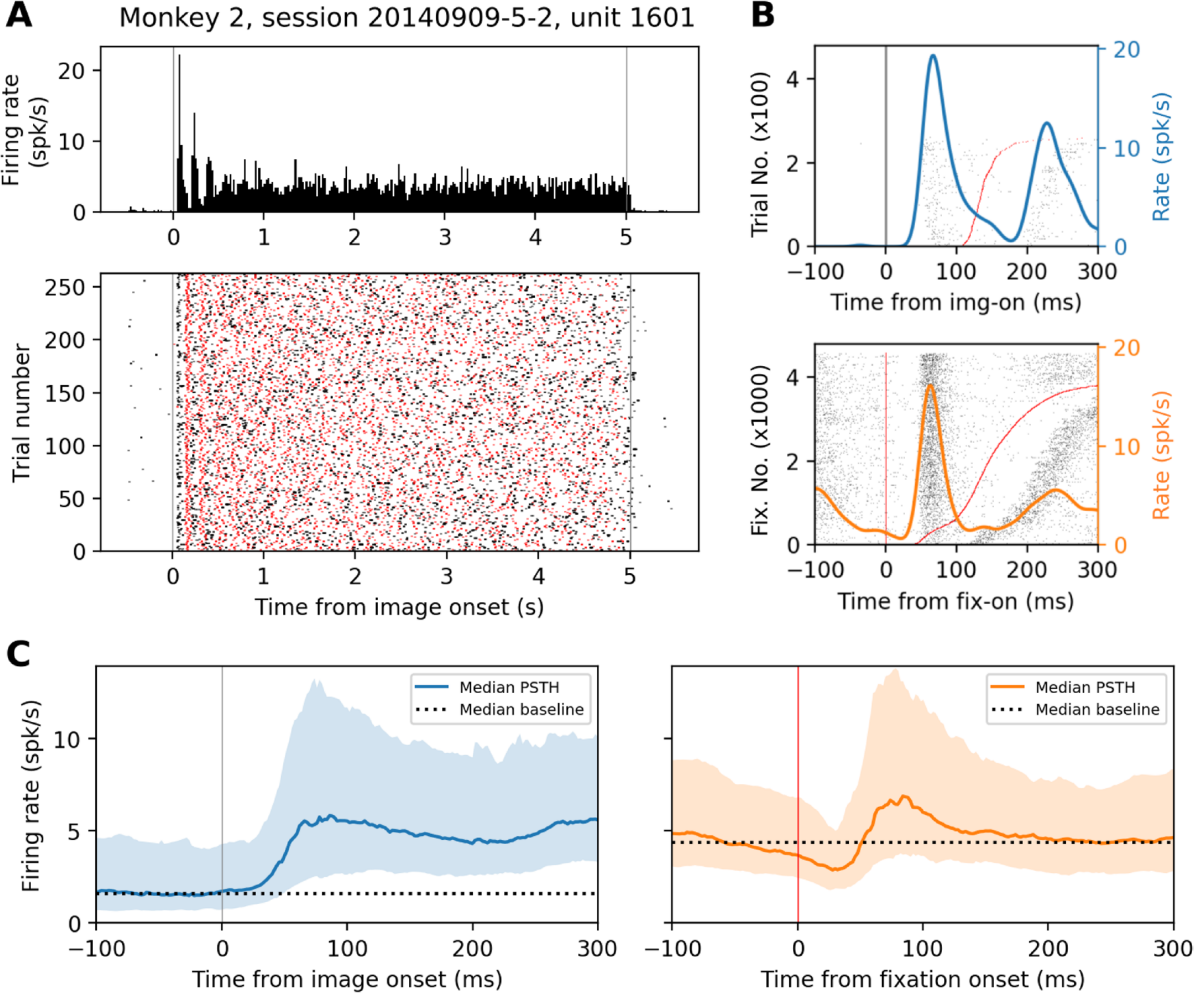
Firing rate modulation in response to image-onset and fixation-onset. (**A**) Raster plot (bottom) representing spike times (black dots) of a V1 neuron of monkey 2 and fix-on times (red dots) in single trials (y-axis), and the corresponding trial-average firing rate (top). The trials are aligned to img-on. (**B**) PSTHs (solid line, scale on the right y-axis) in response to img-on (top panel) and fix-on (bottom panel), for the V1 neuron shown in (**A**). In the background are the raster plots, aligned to img-on (top) or to fix-on (bottom), with the img-on- or fix-on counts scaled on the left y-axis. Red dots mark the following fix-ons, and the trials or fixations are sorted according to the time to the next fix-on. This neuron is classified as a doubly responsive neuron afterwards. (**C**) Grand median PSTH, in response to img-on (left) and to fix-on (right), of all recorded neurons. Shades represent the respective inter-quartile ranges. Horizontal dotted lines indicate the respective medians of the baseline firing rates of all recorded neurons, used for testing significance of single unit PSTH peak heights.

In search for putative differential modulation of neuronal responses in active versus passive viewing, we examine the firing responses of single-units in V1 to img-on (Fig. 2A bottom, black vertical line) or fix-on (Fig. 2A bottom, red vertical lines). Figure 3A shows the PSTH and raster plot of an example single-unit, triggered on img-on for the whole trials. The time locking of the spike activity to the onset of the natural scene images (left gray line) is clearly visible as narrow peaks of the PSTH. Still, successive fixations (red dots) happen at variable time intervals from img-on, leading to visually flat PSTHs during the trial. Figure 3B shows the PSTHs for the same single-unit but either aligned to img-on (top panel) or fix-on (bottom panel) for a duration of 300 ms. This unit shows as a clear response to fix-on as to img-on, with similar latency. These two types of PSTH are computed for all recorded units. Figure 3C shows the grand median of those PSTHs across units. The peak of the median fix-on PSTH is higher than the median img-on PSTH, and it is preceded by a gradual decrease of firing rate continuing from the late part of the preceding fixation.

To detect significant responses of the recorded single-units, we perform, for every single-unit, a significance test of the peak firing rate in each of the PSTHs, comparing the peak rate to the baseline (note that different baselines are used for img-on and fix-on PSTHs (c.f. Fig. 3C); see “Methods” for details). We find that the number of neurons with a significant rate increase for fix-on is 187 out of 217 units (88%), while for img-on we found a lower percentage of responsive units: 116 out of 217 (53%) (Table 1). The median peak rate of the fix-on PSTHs for the fix-on responsive units was 10.52 Hz (inter-quartile range (IQR): 6.13–18.06 Hz) with the median baseline firing rate of 4.90 Hz, while the median peak rate of the img-on PSTHs for the img-on responsive units was 13.89 Hz (IQR: 8.82–22.60 Hz) with the median baseline firing rate of 1.66 Hz. Thus, whereas the magnitude of significant responses is weaker for fix-on than for img-on (in terms of both the absolute firing rate and the difference from the baseline), a larger fraction of neurons exhibit significant responses to fix-on than to img-on.

**Table 1:** Summary of single-unit responsiveness to image and fixation onset.

V1 single-units				
		Fixation onset (fix-on)		Total
		Sign. peak	No sign. peak	
Image onset (img-on)	Sign. peak	113 (52%) [doubly responsive]	3 (1%)	116 (53%) [img-on responsive]
	No sign. peak	74 (34%) [fixation-only responsive]	27 (12%) [non-responsive]	101 (47%)
Total		187 (86%) [fix-on responsive]	30 (14%)	217

We find a considerable overlap of the units that respond in both conditions: 113 units (52%) respond to both trigger events. Only 3 (1%) of the recorded neurons responded solely to img-on and not to fix-on. In fact, 99% of the units that respond to img-on exhibit a significant firing rate increase during fix-on as well. We term these cells *doubly responsive neurons*. Conversely, we find that 74 units (34%) show a significant increment in their firing rate only for fix-on. We term these cells *fixation-only responsive neurons*. In summary, we find that more than half of the neurons respond to both image and fixation onset, while an additional and distinct 34% of the neurons respond only during the free-viewing condition.

**Figure 4:**
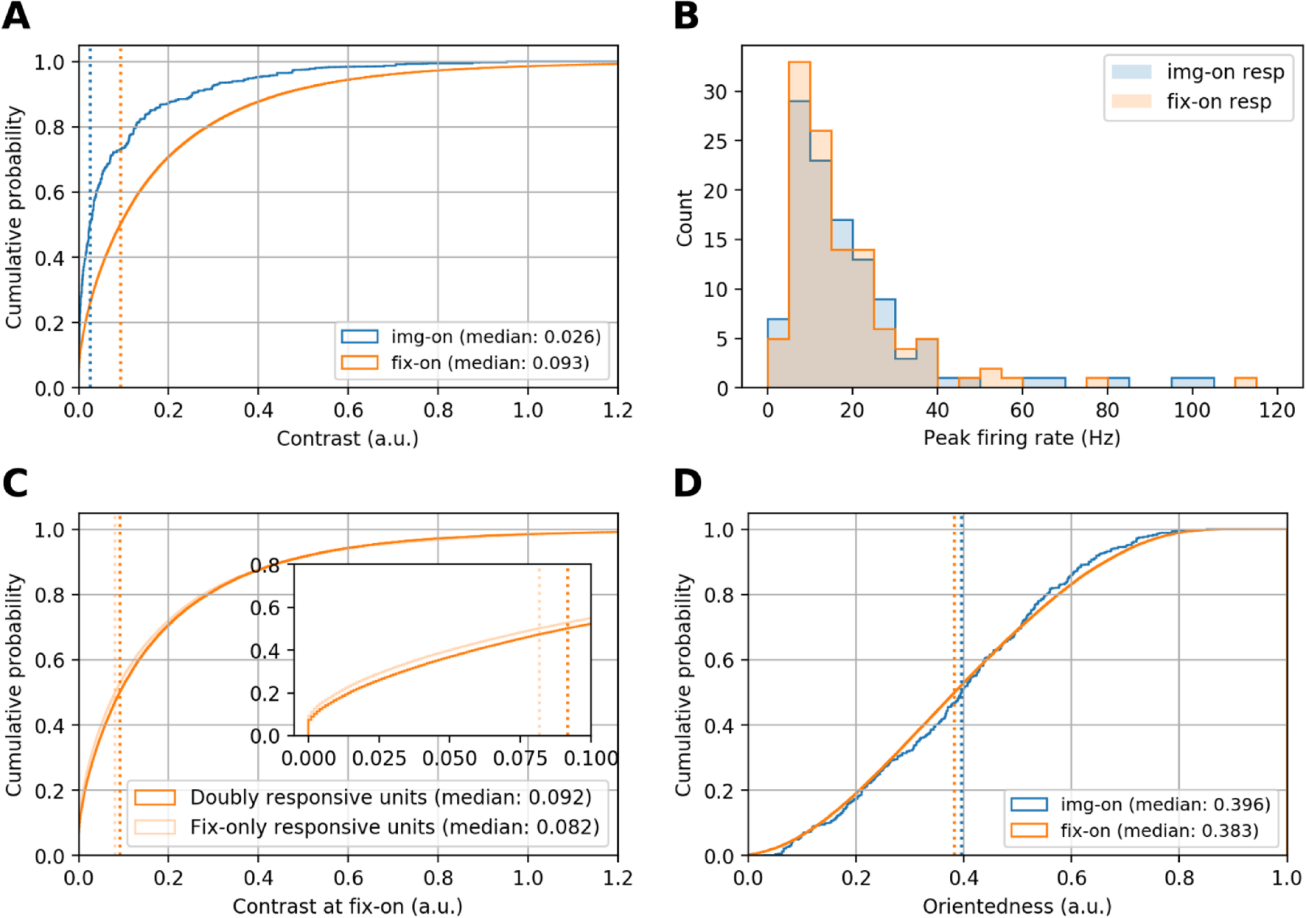
(**A**) Cumulative distributions of contrast of image patches at img-ons and fix-ons, respectively. Vertical dotted lines indicate the respective medians. (**B**) Histograms of the peak firing rates of doubly responsive units in response to img-on and fix-on. (**C**) Cumulative distributions of contrasts at fix-ons, computed separately for fixations while doubly responsive neurons were recorded and for those while fix-only responsive units were recorded. The inset shows the same plot magnified for contrast values from 0 to 0.1. D Cumulative distribution of the orientedness of visual patches at img-ons and fix-ons, respectively.

The large percentage of neurons that respond to fixation onset could be attributed to many factors, e.g., top-down influences on V1, enhancing their activity during active vision, or bottom-up factors such as differences in the statistics of the visual stimuli at img-on and fix-on. Because the animals were allowed to visually explore the entire image during free viewing, their fixation could be guided by salient features and thus increment their neuronal responses^22^. Therefore, differences in the visual stimuli could, in principle, account for the increased responsiveness during free viewing. In particular, visual contrast is one of the most robust features that can increase visual activity^1,23^. To elucidate the influence of the image contrast, we compute for every img-on and fix-on the local contrast (see “Methods”) of the image patch that falls into the receptive field of the recorded neuron and compare the distributions of the contrasts at img-on and fix-on, respectively (Fig. 4A). The two distributions are significantly different (Mann–Whitney U test, p < 10^–16^), with higher median contrast for fix-on than for img-on. This difference is primarily because at img-on the monkeys fixate at the center of the screen, where the local contrast is not necessarily high (e.g., a part of an ambient background), while at fix-on the monkeys mostly fixate on object images, which typically contain higher contrast edges.

The observed contrast difference may indicate that the prevalence of fix-on responsive units is a mere reflection of stronger stimulation of V1 cells by higher contrasts at fix-on than at img-on. If this were the case, the following prediction should hold: doubly responsive units exhibit higher peak firing rates in response to fix-on than in response to img-on, because of the higher contrast at fix-on than at img-on. However, the peak firing rates of the doubly responsive units do not show a significant difference between img-on and fix-on (Fig. 4B; Wilcoxon signed-rank test, p = 0.58). This means that the contrast of the stimulus image patches does not solely determine the spike response magnitude of V1 cells. Therefore, the prevalence of fix-on responsive units is not entirely explained by the higher contrast at fix-on, but further aspects, e.g., active vs. passive vision, impact the firing rates. Furthermore, the fact that fixation-only responsive neurons do not show significant firing rate changes to img-on could be interpreted as that their contrast sensitivity is not high enough to respond to lower contrasts at img-ons. However, we find that the median contrast value for fixation-only responsive neurons is actually lower than that of doubly responsive units (Fig. 4C).

Another aspect of receptive field attributes that potentially impact the firing rates of V1 neurons is stimulus orientation in the image patches. Therefore, we further examine whether orientation strengths in the image patches could be a modulatory factor. We compute the orientation strength as orientedness (see “Methods”). The distributions of the orientedness of patches at img-ons and fix-ons, respectively, are shown in Fig. 4D. We find that the median orientation strengths of these two distributions are not significantly different (Mann–Whitney *U*-test, p = 0.08). These results imply that the higher percentage of cells that exhibit a significant firing rate increase during free viewing is not explained by contrast or orientation strength. This result also argues for the presence of top-down modulations of V1 activity during active vision and a lack of such impact during the classical flashed image paradigm (passive vision).

### Characterization of neuronal activity across cortical layers

**Figure 5:**
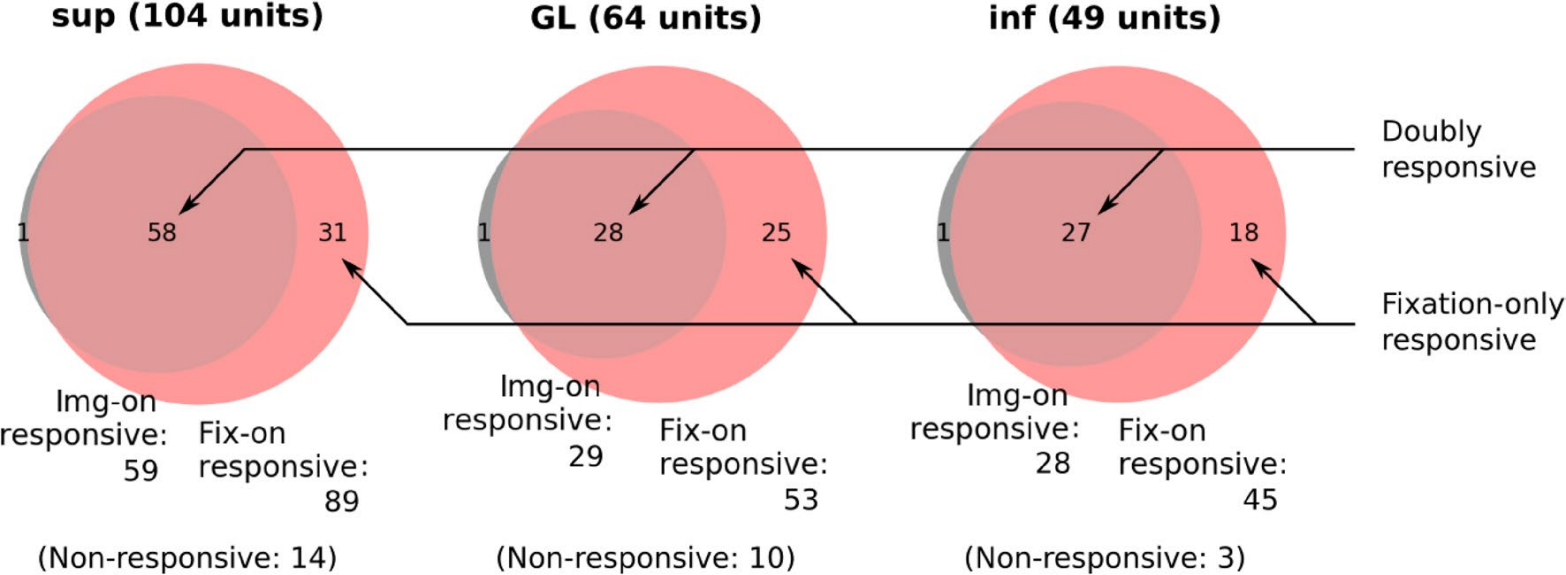
Visual illustration of the results summarized in Table 2. For each layer (sup, GL and inf) a Venn diagram is shown containing in the overlap of the rose (fix-on response units) and gray (img-on responsive units) circles the doubly responsive units. The total number of recorded units is shown in the title of the respective Venn diagram, whereas the numbers of neurons that do not respond (non-responsive) are shown below.

Several previous studies suggested that visual response properties differ across cortical layers^24,25^. Therefore, we next explore the responsiveness, sparseness, and latency of individual cells across layers in V1. The layer information of individual units is derived from a combination of a CSD analysis and histology (Fig. S1). We separate the units into different groups: from granular layer (GL), from supragranular layer (sup), and from infragranular layer (inf). The results of the layer resolved responsiveness are summarized in Fig. 5 and Table 2. In all layers, the corresponding PSTHs mostly have a significant peak for fix-on as compared to img-on (86% vs. 57% in sup, 83% vs. 45% in GL, and 92% vs. 57% in inf).

**Table 2:** Summary of the responsiveness to image and fixation onset, for V1 units resolved into layers.

V1-sup single-units				
		Fixation onset (fix-on)		Total
		Sign. peak	No sign. peak	
Image onset (img-on)	Sign. peak	58 (56%) [doubly responsive]	1 (1%)	59 (57%) [img-on responsive]
	No sign. peak	31 (30%) [fixation-only responsive]	14 (13%) [non-responsive]	45 (43%)
Total		89 (86%) [fix-on responsive]	15 (14%)	104

V1-GL single-units				
		Fixation onset (fix-on)		Total
		Sign. peak	No sign. peak	
Image onset (img-on)	Sign. peak	28 (44%) [doubly responsive]	1 (2%)	29 (45%) [img-on responsive]
	No sign. peak	25 (39%) [fixation-only responsive]	10 (16%)	35 (55%)
Total		53 (83%) [fix-on responsive]	11 (17%) [non-responsive]	64

V1-inf single-units				
		Fixation onset (fix-on)		Total
		Sign. peak	No sign. peak	
Image onset (img-on)	Sign. peak	27 (55%) [doubly responsive]	1 (2%)	28 (57%) [img-on responsive]
	No sign. peak	18 (37%) [fixation-only responsive]	3 (6%) [non-responsive]	21 (43%)
Total		45 (92%) [fix-on responsive]	4 (8%)	49

### Sparseness

**Figure 6:**
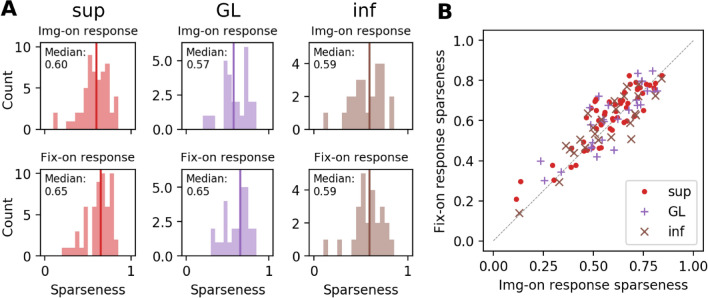
Sparseness in active and passive viewing, resolved into V1 layers. (**A**) Histogram of sparseness values computed for single-units in sup, GL, and inf of V1 with their img-on responses (top) and fix-on responses (bottom). The vertical line indicates the median of the corresponding histogram. (**B**) Scatter plot of the sparseness values of single-unit sparseness for img-on response (x-axis) and fix-on response (y-axis).

After having excluded contrast and orientedness as the most influential factor for the rate differences in active and passive vision, we now explore other aspects of neuronal responses such as sparseness and latency. Several studies have proposed that neurons encode sensory information by activating a specific (sub-)set of cells when responding to a stimulus. This sparseness was suggested to make it easier for higher areas to learn structure in the data^26^. Here, we compute the lifetime sparseness, i.e., the responses of a particular unit to different stimuli. In our case, the different stimuli are given as the image patches at every fixation, or image onset, respectively. Thus every unit will be characterized by its sparseness by Eqs. (1) and (2) (see “Methods”)^18,27^. We compute the sparseness S based on the spike counts within 100 ms after trigger onset, for every single-unit, separately for img-on or fix-on onset events. The distributions of sparseness for all V1 cells resolved by layers are shown in Fig. 6A. While the median sparseness value is greater for fix-on than for img-on in GL and sup, this difference is significant only for sup (p = 0.00248, Wilcoxon signed-rank test). Thus, supragranular neurons respond to fewer stimuli during active vision than during passive vision (Fig. 6).

### Latency

**Figure 7:**
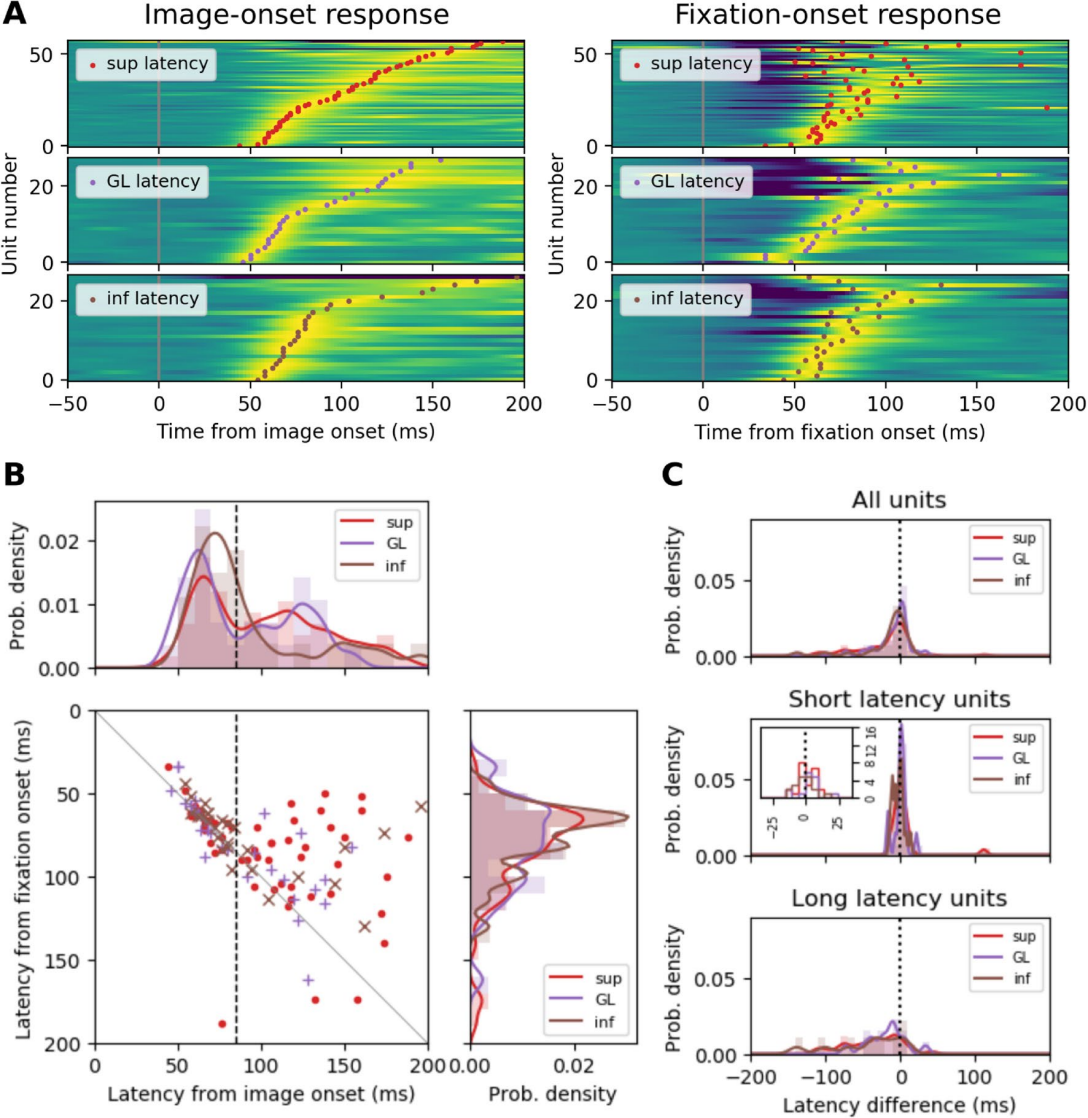
The difference between the response latencies in the passive and active viewing conditions, resolved into V1 layers. (**A**) The normalized PSTHs of individual units (background color) in the 3 layers of V1, and the latencies of PSTH peaks (dots). The blue-green-yellow color gradient in the background corresponds to negative-zero-positive normalized firing rate. Note that the same ordering (i.e., the ascending order of the response latency to the img-on) is used for the units in the identical layer (left and right panels). (**B**) Scatter plot of the response latencies of individual units to img-on (x-axis) and fix-on (y-axis), together with the marginal distributions of the latencies at the right (for the img-on response latency) and the top (for the fix-on response latency). The vertical dashed line indicates the separation between short-latency units and long-latency units. (**C**) Distribution of the difference between the img-on response latency and the fix-on response latency for each of the 3 layers. Top: distributions computed from all units. Middle: distributions computed only from the short-latency units. Inset shows a close-up view of the middle part of the distributions. Bottom: distributions computed only from the long-latency units.

Next, we examine the response latencies, i.e., the timings of the peak of the PSTHs of each unit in the passive and the active condition. To compare the img-on and fix-on latencies for an identical set of units, we examine the latency differences for the doubly responsive units only. We find that while several neurons exhibit a similar latency in both conditions (Fig. 7A), the mean latency is much shorter in the active than in the passive condition and the width of the distributions becomes much narrower in the active condition. 69 out of 113 (61%) units shorten their latency, and mainly the long latency units (> 80 ms) during passive vision shorten their latency considerably. To quantify the changes in the two conditions, we show the latencies in a scatter plot (Fig. 7B) and form the marginal distributions of the latencies in the active condition (top) and the passive condition (right). A majority of the entry points for fix-on are above the diagonal. The distributions of the latencies for the different layers (the marginal distributions) show a shortening in the mean and a reduced variability for the fix-on latencies compared to the img-on latencies.

In response to img-on, the short-latency units show a systematic delay in their response latency across the layers: GL is first, followed by sup, and inf is last (Fig. 7B, top), as expected from the canonical cortical microcircuit^28,29^. The latencies are significantly different between the short-latency units in GL and inf during the passive vision, but during active vision, this difference disappears. These results indicate that, during active vision, neuronal processing is faster than in the passive condition, and the different layers seem to adapt their latencies to an overlapping timing. The latter point suggests a relation to a previous report which showed that during active vision, eye movement-related signals modulate the precise timing of the activity of neurons in V1^14^.

### Control for contrast difference

**Figure 8:**
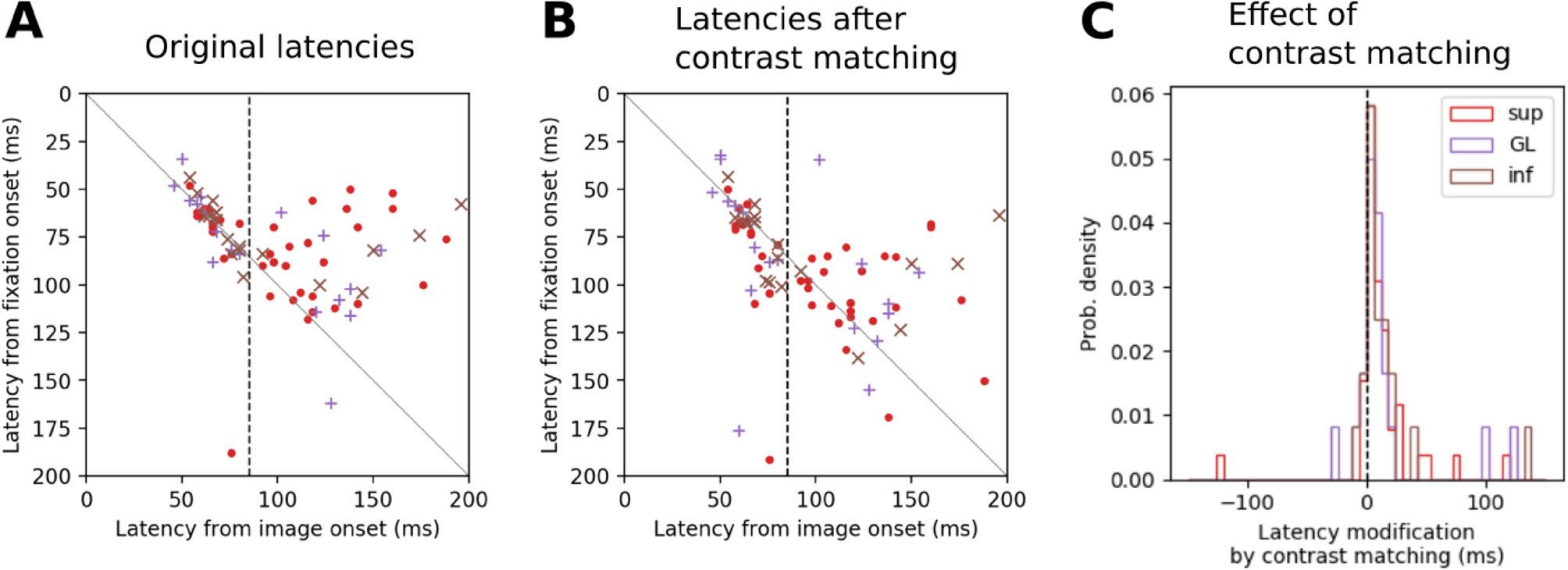
Effect of stimulus image contrast on fix-on response latency. (**A**) The same scatter plot as in Fig. 7B, but only with a subset of units for which the receptive field positions were identified. (**B**) A scatter plot for the same units as in (**A**), but with fix-on response latencies recalculated using only fixations with image patch contrasts comparable to ones at img-ons (i.e., contrast matching between fix-ons and img-ons). (**C**) Histogram of the differences between the original and contrast-matched fix-on response latency.

A possible explanation for the observed latency shortening during active vision could be that the shortening results from higher contrast at fix-on, as it has been reported that stimulus contrast strongly affects the response latency of V1 neurons, i.e., higher contrast leads to shorter latency^30^. Therefore we next compare img-on and fix-on response latencies after matching the stimulus contrasts in the two conditions. For doing that, we focus on the latencies for the subset of neurons whose receptive fields were determined and recalculate their fix-on latencies using only the fixations on image patches that have comparable contrasts to those at img-on. Then we compare the obtained latencies of the contrast-matched fix-on responses against (the original) img-on response latencies (Fig. 8B). Although this contrast-matching procedure generally results in a prolongation of the latencies (Fig. 8C), the main observation of the latency shortening for the long latency units still clearly holds (Fig. 8B) irrespective of the contrast. This result indicates that even when the high contrast at fix-on compared to img-on contributes to a latency shortening during the active vision to some extent, this effect is not strong enough to explain the degree of the considerable latency shortening observed for the long latency units.

**Figure 9:**
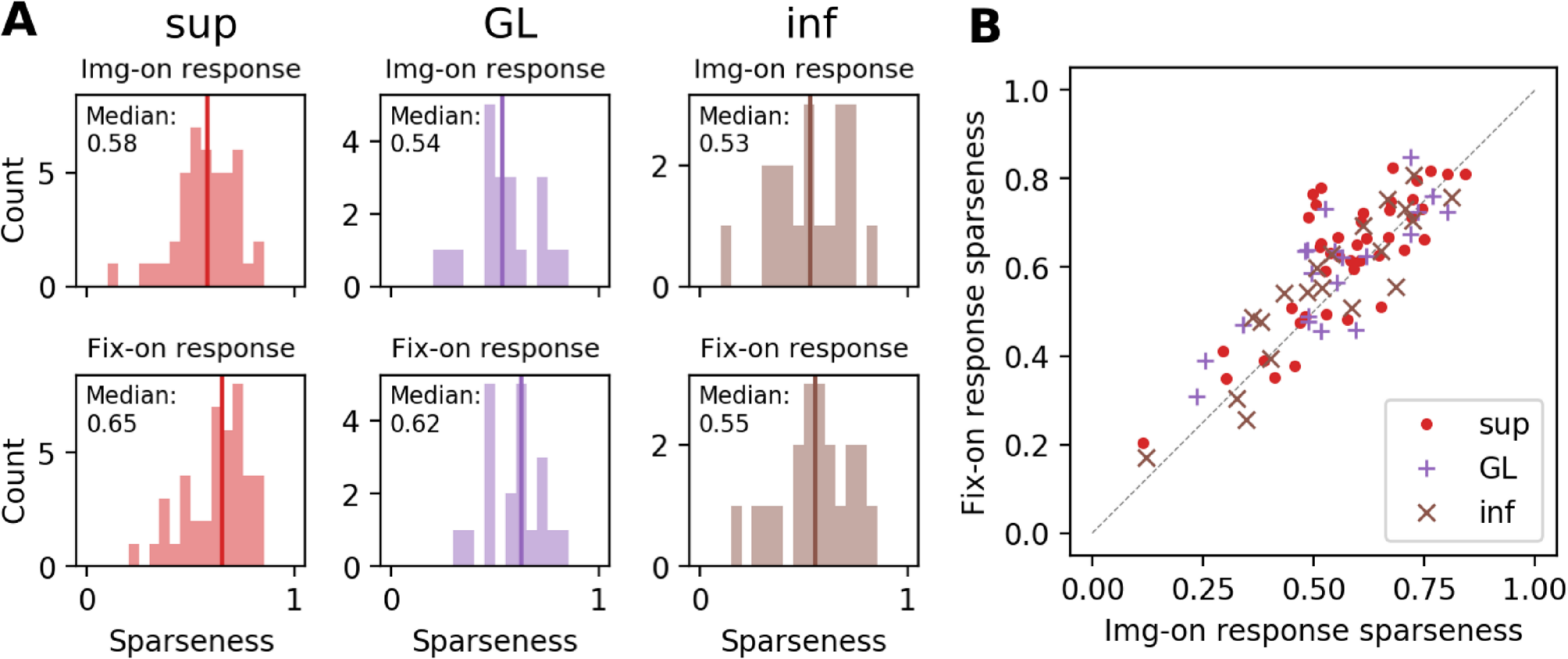
Comparison of sparseness between img-on and fix-on after contrast matching. Here the sparseness values are calculated, differently from Fig. 6, only for a subset of units for which the receptive field positions were identified. (**A**) Histogram of sparseness values computed for single-units in sup, GL, and inf of V1 with their img-on responses (top) and contrast-matched fix-on responses (bottom). The vertical line indicates the median of the corresponding histogram. (**B**) Scatter plot of the sparseness values of single-unit sparseness for img-on response (x-axis) and contrast-matched fix-on response (y-axis).

Sparseness can also be influenced by the difference in the contrast, or more concretely, in the distribution of the contrast values between img-on and fix-on. Therefore we also compare img-on and fix-on response sparseness after matching the stimulus contrasts in the two conditions. We recalculate fix-on response sparseness for the neurons with an identified receptive field, using only the fixations on image patches with contrasts comparable to those at img-ons. Then we compare the obtained contrast-matched fix-on response sparseness to the img-on response sparseness of the identical neurons (Fig. 9). Consistently with the results without contrast matching (Fig. 6), the fix-on response sparseness is considerably greater than the img-on response sparseness in sup and GL, with a significant difference only for sup (p = 0.00234, Wilcoxon signed-rank test). Thus, the observed sparseness enhancement in sup during active vision is not an artifact caused by the contrast difference between img-on and fix-on.

## Discussion

This study aimed to test the hypothesis that neurons in V1 change their activity due to actively exploring visual scenes, in contrast to passive stimulation. Here we studied the responses of single neurons during free-viewing (active) and to image presentations while fixating (passive). We considered the onset of the presentation of a natural scene as the passive condition and the following free-viewing condition as active. We characterized and compared the responses in the two conditions regarding responsiveness, response latencies, and lifetime sparseness. To relate our findings to potential top-down mechanisms, we performed these analyses separately for the V1 layers.

We found that most V1 neurons respond significantly in the active condition but less in the passive condition, i.e., more neurons change their activities during active vision. On the other hand, the lifetime sparseness of the neurons is enhanced during active vision, and thus responses become more distinct compared to passive vision. The response latencies during active vision become shorter and more aligned in time across neurons than in the passive condition. In particular long-latency neurons considerably shorten their latencies during active vision. These findings hold for all layers in V1, but for supragranular cells, we found a considerable increment in sparseness during active vision. Thus, the active and the passive viewing conditions reflect two distinct sensory processes.

We also found that the average duration of fixations during active vision is similar to the average latencies to the first saccade after img-on. Similar results were reported in human and other primate species^16,31^. Both the latency to the first saccade and the fixation duration during free viewing would suggest a minimal time for visual processing during which the visual system inhibits further saccadic movements until processing of the current stimulus is completed^32^. Nonetheless, we find that the distribution of fixation durations is broader than the first saccade latency distribution. This larger variability of fixation durations may be due to two distinct behavioral modes found during free-viewing in monkeys^15^. It was found that during freely viewing a natural scene, the monkey first scans the visual environment by visiting objects just once with short fixations (ambient mode), and then in a second period fixates longer and several times on an object (local mode).

### Responsiveness and top-down mechanisms during active vision

In our study, V1 neurons show greater responsiveness in active compared to passive vision. One study by Gallant et al.^33^ showed that 58% of V1 cells were responsive to visual fixations on natural scenes, an active sensing situation. Their study considered a cell responsive if they observed a specific change in the firing rate in 15-ms bins of the PSTH, but mentioned that the recording time in their experiment might have limited their ability to obtain reliable responses. In contrast, Tang et al.^34^ used calcium imaging to record monkey’s V1 neurons while stimulated with small flashed patches of natural images presented in their receptive field. They found that only 0.5% of neurons responded strongly to the onset of this passive condition. Although these results cannot be quantitatively compared to ours because the experimental setups are different, our results are qualitatively consistent with these previous reports. We also showed that differences in basic receptive field properties, such as contrast or stimulus orientation, do not fully account for the enhanced responsiveness, but this must be due to other mechanisms such as top-down modulatory mechanisms. MacEvoy et al.^19^ reported that responses of V1 neurons to an optimal bar stimulus differ depending on whether the stimulus was flashed or brought into the receptive field via saccades. Ahissar and Assa^21^ showed that during active vision, self-initiated eye movements cause a change of visual input on the retina, which was interpreted as a putative engagement of closed-loop modulatory mechanisms. Indeed, Ito et al.^14^ found evidence for such a top-down mechanism, reflected as saccade-locked LFP modulations in V1, which influence the timings of the spiking activity induced by visual input. This kind of eye movement-related top-down modulation of V1 activity is a potential mechanism underlying the enhanced responsiveness in the present study.

Other studies have reported top-down saccade-related modulations, such as saccadic suppression and corollary discharge signals in V1 neurons^35,36^. Paradiso et al.^37^ showed that the local field potential (LFP) signal carries information about the onset time of fixation directions of saccades in a forced-saccade paradigm. Rajkai et al.^38^ showed that current source density patterns of a flash stimulus are different from those occurring during voluntary cascades. In the latter case, neural activity synchronized to fixation onset suggested that a brief period of cortical excitation follows each fixation, as also shown by Ito et al.^14^. Also, Gawne and Woods^39^ demonstrated that the responses to the saccade-induced presentation of a stimulus within a neuron’s receptive field were typically suppressed by the presence of a stimulus in the receptive field before the saccade. In addition, Burr et al.^40^ showed that the spatial frequency of grating presented during force saccade differentially affects magnocellular and parvocellular pathways. Recently Niemeyer and Paradiso^41^ found that in a forced-saccade paradigm where small static Gabor patches were presented against a dimmed natural image, contrast sensitivity was reduced up to 25% compared to a fixation paradigm, resulting in decreased V1 responses. They concluded that these reductions in V1 neurons during natural vision result from fast adaptation on one fixation to the next. Although these reports in post-saccadic excitability and contrast sensitivity can contribute to the changes in responsiveness on fix-on, these studies are not readily comparable to our current study because they were performed using simple stimuli such as gratings and obtained under forced-saccade conditions.

### Response timing during active vision

We found a shortening of the response latencies during free-viewing. Several previous studies^24,25^ have shown that top-down influences on V1 are not seen in the initial response to visual stimulation, but the response was found in a later period around 100 ms after stimulus onset. As these studies employ a passive stimulation paradigm, the neurons contributing to such late responses should correspond to the long latency units in our present study, i.e., the neurons that exhibit latencies later than 80 ms after image onset. We found that primarily the long-latency neurons shorten their response latencies during active vision. This suggests that top-down related processes, implicated by the long response latency in the passive vision, are facilitated or different in the active viewing condition resulting in a shortening of their latency.

The fact that there is a shortening of the response latencies during active vision may be taken as a strong indication that the system is prepared for the upcoming visual input, i.e., expecting the stimulus at a specific time because a saccade was performed before. This process is consistent with active sensing^42^, although the original expression comes from machine perception, see^43^, which is based on the concept that the brain actively explores the environment by its own motor acts to get sensory input. This relationship between a motor act and sensory activity has already been reported in several studies^44,45^ and has also been observed in other modalities such as olfaction^46^, audition^47^, and somatosensation^48^. This principle in which motor action modulates visual sensory activity indeed occurs in humans but also in other animals such as rats and primates, fish and bats, which suggests this as a general mechanism in visual perception^49–52^.

Ito et al.^14^ described a potential mechanism for shortening the latencies during the active condition. In that study on Cebus monkeys freely viewing natural scenes, it was found that the local field potential (LFP) in V1 exhibits oscillatory modulations which are locked to saccade onset. These LFP modulations, however, happen after fixation onset, and the spikes elicited by visual input (i.e., upon fixation) lock to the phase (around the trough) of the LFP. The latency of the LFP trough exhibited a maximum at about 75 ms from fixation onset. This fact is consistent with our findings that the response latencies in active vision are bound to about the same latency, thus being a putative mechanism that accounts for this observation. Thus, we suggest that a top-down mechanism related to eye movements induces an LFP modulation in V1, which alters the spike timing of multiple neurons to a specific time epoch.

### Sparseness enhancement during active vision

It has been proposed that the striate cortex generates a sparse and dispersed code that is highly efficient for storing information and associative learning^53^. Indeed, several studies have shown that cells in V1 responded sparsely to natural images^34^. Our study found that cells in V1 increase their sparseness from the passive to the active condition, i.e., cells respond more strongly and to a lower number of image patches than to the (flashed) image onset. Weliky et al.^54^ found similar sparseness values to our study. Tang et al.^34^ employed large-scale two-photon calcium imaging to examine V1 cell responses in awake monkeys while being flashed with 4 deg patches of natural images. They reported an average lifetime sparseness of somewhat lower values (0.49 and 0.41) than ours for each monkey. The differences in sparseness between this study and ours can be explained, in part, by the fact that the visual stimuli in our study are larger^18^.

It might seem contradictory that both our responsiveness and lifetime sparseness measures are higher during active vision than passive vision. This impression might stem from an interpretation that our responsiveness is a proxy of population sparseness, but such an interpretation is not correct. We define *responsiveness* as the fraction of neurons responding significantly to img-on or fix-on, where we did not distinguish the identity of stimuli eliciting the responses. Therefore this measure differs from population sparseness in that our responsiveness does not consider whether or not multiple neurons were activated simultaneously by the same stimulus. Lifetime sparseness, in contrast, evaluates the fraction of stimuli eliciting a response in a single neuron, and in this calculation, the magnitude of response is not considered. Irrespective of the degree of sparseness, neurons are considered as responsive if they responded strongly enough. Thus, these two measures are independent of each other.

### Layer specific responses

This study manifests active sensing by self-recruiting visual input by eye movements to particular visual field locations. This process implies the possibility of top-down anticipatory or modulatory mechanisms, which may be specific for different cortical layers^55^. Anatomical studies have shown that cortico-cortical connections to V1 from higher cortical areas target the supragranular and infragranular layers of V1^56–58^. We found that the long-latency units of these layers primarily shortened their response latency during active vision, which resulted in unimodal strongly overlapping distributions of the three layers in response to fix-on. In contrast, these three distributions had different peak timings with sup and GL showing bimodal distributions during passive vision. This suggests that feedback input to V1 during active vision leads to a modulation of V1 activity and finds its expression in a change of the peak latencies that strongly overlap across layers during active vision.

We also found a substantial increment in sparseness during active vision for the responses of the neurons in the supragranular layer compared to passive vision. This finding can be interpreted as the ability of the cortex to exploit predictive aspects of an image while visually exploring visual scenes that can hardly be present in the case of a flashed image. Some models of predictive processing in sensory cortices postulate that the prediction error is computed in a cortical column and is then fed forward to higher cortical areas by neurons of the supragranular layer^28,59^. Increased predictability of visual stimuli during active vision (i.e., self-acquired visual information) than passive vision (flashed stimuli) should result in a smaller prediction error and, therefore, in the increased sparseness of these neurons as a result of these neurons predictive suppression of their responses. Thus, our findings are consistent with the hypothesis of predictive information processing in the visual cortex.

## Conclusions

In this work, we compare active vision, i.e., the situation of free-viewing, in comparison to passive viewing, where a stimulus is flashed while fixating. We observed several differences during the two conditions (higher responsiveness and sparseness and latency shortening in active vision) and interpreted those as the consequence of top-down influences present during active vision, but not or less during passive vision. Active vision incorporates active sensing, i.e., visual input is actively acquired through saccadic eye movements. The brain itself decides the positions of fixations before executing the saccade, which enables predictive input. Top-down input to V1 supports more distinct and more timely adapted activities, effectively enabling coordinated processing.

Several studies have started to explore the underlying mechanisms of active sensing, i.e., coupling of motor acts and sensory inputs. Devia et al.^60^ reported comparable results to those reported here in EEG signals. They found that the evoked potentials to visual fixations doubles in magnitude and differs in shape compared to flashed stimuli. They also found that the ERP to saccade onset was as large as the ERP to fixation onset, however with an early component that preceded the visual input, suggesting a motor modulation with saccades. Barczak et al.^61^ showed excitability and response amplification in V1 cells immediately after fixation onset, transiting to suppression up to the time of the next saccade. The latter stems from a phase reset of ongoing neuronal oscillatory activity. Such self-initiated general stimulus acquirements have behavioral consequences. Carcea et al.^62^ showed that after self-producing auditory stimuli, rats improved their auditory discrimination capabilities, as observed in decreased detection thresholds and through tuning of their psychophysics curves, and Concha-Miranda et al.^63^ showed that rats improved their performance in a visual discrimination task when the animals self-initiated the onset of the stimulus.

Our findings presented here also clearly suggest that neuronal mechanisms present during free-viewing, i.e., active vision, may become apparent only during active sensing engagement. Considering that most of what we learned here about the visual system is very different from experiments where the experimenter controls stimuli and behavior, we stress the need further to explore potential differences between the classical and natural paradigms.

## Methods

### Animal preparation

Two female macaque monkeys (*Macaca fuscata*), referred to as monkeys 1 (body weight: 5.2 kg, age: 4 years) and 2 (body weight: 7.1 kg, age: 7 years), were used in this study. A head restraint and two recording chambers for the electrophysiological recordings, one over V1 and the other over the inferior temporal cortex (IT), were implanted in the skull of each monkey (Supplementary Fig. 1A). The recording position in V1 was adjusted such that the receptive fields of the recorded neurons were close to the fovea. The recording position in IT was in a range of 0.5–9.0 mm (for monkey 1) or 2.6–11 mm (for monkey 2) anterior from the auditory meatus and included the lateral convexity of IT cortex and the superior temporal sulcus (STS). The detailed method of the surgery was described in Ito et al.^15^. Briefly, the surgery was performed under full anesthesia by inhalation of 1–3% isoflurane (Forane, Abbott Japan, Tokyo, Japan) in nitrous oxide (70% N_2_O, 30% O_2_) through an intratracheal cannula. An antibiotic (Pentcillin, Toyama Chemical, Toyama, Japan; 40 mg/kg by intramuscular injection) and an anti-inflammatory and analgesic agent (Voltaren, Novartis, Tokyo, Japan, 12.5 mg; Ketoprofen, Nissin Pharmaceutical, Yamagata, Japan, 0.6 mg/kg; both administered by intramuscular injection) were given immediately after the surgery and continued during the first postoperative week. After 1–2 weeks of recovery, a scleral search coil for the measurement of the eye position was implanted in the left eye under the same anesthesia as mentioned above. After the recovery from the surgery, we trained the animals for behavioral tasks (see “Behavioral tasks'' section) for around 2 weeks. We then started the recordings by acutely inserting 2 linear-array electrodes (see “Recording system and electrodes”) into V1 and IT. The recordings from IT were not analyzed in the present study (except for examination of the object selectivity of IT neurons, to be used for the selection of object images to compose stimulus images, as described later.) All experiments were performed in accordance with the guidelines of the National Institute of Health (1996) and the Japan Neuroscience Society, and were approved by the Osaka University Animal Experiment Committee (certification No. FBS-13-003). We did not register any protocol before the experiment.

### Behavioral task

The monkeys sat in a chair with its head fixed. An LCD monitor for stimulus presentation (FlexScan EV2736W-FS, EIZO, Ishikawa, Japan) was placed 57 cm away from the eyes of the monkey. The two monkeys were trained to perform an eye calibration task, a fixation task, and a free-viewing task (detailed task descriptions are given later). We define a 'recording session' as a set of trials for one particular task. The typical sequence of the recording sessions on a day was (1) an eye calibration task session, (2) several fixation task sessions to determine the recording depth of the electrodes, (3) a fixation task session to examine the object selectivity of IT neurons, (4) an eye calibration task session, (5) free-viewing task sessions, (6) an eye calibration task session, and (7) a fixation task session to examine the orientation selectivity of neurons in V1.

#### Eye calibration task

The eye calibration task was performed at the beginning of the recording on each day. In most cases, this task was also performed before and after the main free-viewing task sessions to ensure the precise estimation of the eye position during the free-viewing. In each trial of the eye calibration task, a square fixation point (0.2°) was shown at one of 9 positions on a 3 × 3 rectangular grid extending 38° width × 28° height. The grid covered an area slightly larger than the stimulus images used in the free-viewing task, to ensure a good calibration even at the boundaries of the images. The monkeys were required to fixate within 0.5° radius around the fixation point for 800 ms to complete a trial. After each trial, the monkey received drops of water or juice as a reward. The task continued until 3 successful fixations were completed for each of the 9 positions. The average vertical and horizontal voltage values from the search coil system during the fixations were used to calculate the transformation of the measured voltages into the gaze positions.

#### Fixation task

A conventional fixation task was performed in order to determine the recording depth of the probe that picked up the signal along the linear array electrode, based on a current source density analysis of visually evoked local field potentials^24^ and to quickly examine the stimulus selectivity of IT neurons. The task started with a presentation of a fixation spot (0.2° square) at the center of the monitor screen, followed by the presentation of object images used in the free-viewing task (see “Visual stimuli” section) for 200 ms each, with a 200 ms inter-stimulus interval. Typically, 6 object images were presented sequentially in one trial.

#### Free-viewing task

A trial started with the presentation of a fixation spot (0.2° square) at the center of the monitor screen (Fig. 1). After the animals fixated the fixation spot for 0.5 s, the spot was turned off and a stimulus image was presented for free-viewing (see “Stimulus preparation” section). After the onset of a stimulus image, the animals were allowed to move their eyes freely, but they were not allowed to direct their gaze outside the boundaries of the image. If they did, the trial was aborted with no reward. If the monkeys succeeded in keeping their gaze within the image boundaries for 5 s, the image was turned off and then, after a 0.5 s delay, drops of water or juice were delivered as a reward. The inter-trial interval was 1.5 s for monkey 1 and 2.0 s for monkey 2, depending on the monkeys’ patience with waiting for the next trial. The free-viewing task session continued as long as the motivation of the animals lasted (typically more than 30 min). Usually, there was one free-viewing task session conducted on a day.

### Stimulus preparation

The stimulus images for the free-viewing task were generated by placing 5 object images (2° in diameter on average) on a 34.8 × 26.1 deg^2^ background image. The object images were taken either by one of the authors or drawn from the Microsoft image gallery (used to be available on Office Online: https://www.office.com) with permission from Microsoft. The background image was one of 67 natural scene images, taken either by one of the authors or drawn from the Natural Image Database of Center for Perceptual Systems, the University of Texas at Austin (http://natural-scenes.cps.utexas.edu/db.shtml^64^; and the McGill calibrated color image database (http://tabby.vision.mcgill.ca/^65^, with permission from the copyright holders.

We prepared a set of 64 object images and used this set for the initial survey of the object selectivity of IT neurons in the fixation task session. Then, for every free-viewing task session, 20 object images that elicited the largest multi-unit response rates were selected from this set. To create one stimulus image, 5 objects were randomly chosen from the 20 objects and were placed on a randomly chosen background image at pseudo-random positions, with a constraint that the distance between any pair of the objects was larger than 4° thereby ensuring that the objects did not overlap with each other. We generated 60 stimulus images for one free-viewing task session. They were presented in a pseudo-random order. Each image was presented 3–5 times depending on the total duration of the session.

### Recording system and electrodes

The tasks were controlled by a custom-made program on a programmable logic controller (KV5000, KEYENCE, Osaka, Japan). The eye positions were recorded with a scleral search coil system (DSC2000, SANKEI, Japan). The extracellular neuronal activity was recorded using linear array electrodes with 24 channels at 100 μm intervals (V-probe, Plexon Inc., Texas, USA), amplified and band-pass filtered (0.7 Hz–8 kHz) using a commercial amplifier (Plexon Inc., Texas, USA). The neuronal activity and the eye position data were acquired through an A/D board (National Instruments, Texas, USA) at a sampling rate of 20 kHz and stored on hard disk drives for offline analyses.

### Eye event detection

The detailed method of eye event detection is described in Ito et al.^15^. Briefly, we first estimated the velocity and the acceleration of the eye movements by computing the temporal derivatives of the eye position data using Savitzky-Golay filters^66,67^. Then, we collected the time segments throughout which the eye velocity and the acceleration were above 30°/s and 8000°/s^2^, respectively, as potential saccade periods. We discarded the segments containing noise or artifacts based on velocity, acceleration, duration and amplitude criteria. The remaining segments were identified as saccade periods, and the start and end times of each segment were registered as saccade onset and offset, respectively. We made no discrimination between microsaccades and normal saccades.

Intervals between two successive saccades were considered as potential fixation periods. Those intervals with a gaze shift exceeding 1° were discarded. The remaining intervals were identified as fixation periods. The offset of the preceding saccade was registered as fixation onset, and the time duration from the fixation onset to the onset of the following saccade was registered as fixation duration.

### Spike sorting

Extracellular action potentials (or spikes) were first processed by using the Kaneko-Tamura-Suzuki (KTS) spike sorting algorithm^68^. The KTS sorter utilizes signals from multiple electrodes to track gradual changes in the spike amplitudes over time. During the course of spike sorting we noticed that the change in spike amplitudes was larger than originally expected by the KTS sorter. This may be due to multiple factors stemming from our experimental setup, such as the size of the electrode, the acute penetration, the awake behaving animal, and so on. The unexpected amplitude change resulted in the following abnormalities in the sorted spike trains: (i) gradual or abrupt changes in the spike amplitudes over time and (ii) gradual or abrupt changes of the firing rates of the sorted units. These findings claimed for further inspection of the single-units that were identified by the KTS sorter, which we term as KTS units. It revealed that a part of the KTS units seemed to be contaminated by spikes belonging to other units. Thus, post-processing for teasing out those spikes was necessary to improve the sorting quality. This was performed in the following steps: (1) *spike extraction*, (2) *re-clustering*, (3) *cluster merging*, and (4) *spike train segmentation*. Details of each step are as follows.

#### Spike extraction

The spike waveforms of the KTS units were extracted from the high-pass filtered (> 500 Hz, fourth-order Butterworth filter) raw extracellular recordings. The obtained waveforms were up-sampled to 200 kHz via cubic interpolation, and time-aligned on their waveform minima.

#### Re-clustering

Principal component analysis (PCA) was applied to the obtained spike waveforms of every KTS unit and the spikes were re-clustered in the PC space (of the first 3 PCs) up to 3 clusters by using the Gaussian mixture model method. These clusters were identified as single-unit activities (SUAs). To avoid multi-unit contamination, we rejected clusters with a refractory period violation (threshold at 1.2 ms) of their inter-spike intervals (ISIs) above 0.1% of all ISIs.

#### Cluster merging

In the preceding step, spikes of an identical neuron could have been separated into multiple clusters according to the issue (i) above. To avoid over-disaggregation of SUAs by this procedure, we computed pairwise cross-correlations between cluster-averaged spike waveforms for all pairs of clusters from a single KTS unit and merged every pair of clusters with R^2^ > 0.95 into one cluster. The validity of each cluster merging was checked by three independent human inspectors, and the merging was approved only when all three inspectors confirmed the validity. We further checked the similarity of the waveforms of KTS units in a single channel and merged them into one unit if they fulfill the above-mentioned criteria. The resulting clusters were grouped as separate SUAs.

#### Spike train segmentation

The obtained SUAs typically showed gradual or abrupt variations in their firing rates, i.e., issue (ii) mentioned above. To base our analysis only on spike train data with stationary firing rates, we applied a rate change point detection method in the time-resolved firing rate of the SUA on each newly grouped SUA of a free-viewing task session and identified the time segments during which no firing rate change was detected. Only the data of the longest segment of each single-unit was used for spike data analyses. The change-point detection was performed in the following manner: first, for a given single-unit, we counted the number of all its spikes in every free-viewing trial (called ‘trial spike count’); second, we took the first 30 trials of the session and compared the distributions of the trial spike counts for the first 15 trials and the last 15 trials, by performing a Kolmogorov–Smirnov test on the two distributions to obtain a *p*-value; third, we repeated this for each and every successive 30 trials (called ‘trial blocks’) in the session, to obtain a series of *p*-values; fourth, we applied the false discovery rate control (Benjamini–Hochberg procedure at an alpha level of 0.05^69^ to the series of *p*-values, and identified a change-point at the middle of each trial block (i.e., the border between the first and the last 15 trials of the trial block) with a *p*-value below the alpha level.

### Single-unit classification

To infer the electrode insertion depth in relation to the cortical layers, we performed a current source density (CSD) estimation using the local field potential (LFP) signals recorded simultaneously with the spiking activity. The LFPs were obtained by band-pass filtering (fourth-order Butterworth filter) the raw extracellular recordings in the frequency range of 1–100 Hz. The CSD was estimated by applying the inverse CSD method^70^ to the parallel LFP signals recorded at the 24 probes on the linear electrode array (100 μm electrode intervals, spanning 2.3 mm) inserted perpendicularly to the brain surface. In most free-viewing task sessions, the image-onset triggered average of the CSD signal in V1 exhibited a clear signature of the sink-source pair (Supplementary Fig. 1C) that had been reported to mark the boundary between layers 4c and 5^24,25^. In some recording sessions we recognized a second sink-source pair in a location much deeper than the first sink-source pair, which we identified as V2 activity beyond the white matter (Supplementary Fig. 1B,C). According to these CSD signatures, the recording sites between 0.4 and 0 mm below the 4c/5 boundary were assigned to the infragranular layer, sites between 0 and 0.4 mm above the boundary were assigned to the granular layer, and sites more than 0.4 mm above the boundary were assigned to the supragranular layer (Supplementary Fig. 1C). Single-units recorded at these three sites were considered as V1 units. Single-units recorded at the sites more than 1 mm below the 4c/5 boundary were considered as V2 units, which were most likely located in the infragranular layer of V2 and therefore not included in the analyses in the present study.

In addition, we determined the receptive field (RF) location for the multi-unit activity recorded from the channels corresponding to the V1 layers, and found that all RFs were smaller than 2° and located not further than 2° from the fovea (Supplementary Fig. 2). Since we inserted the linear electrode array perpendicular to the brain surface and sampled single units mostly in an identical cortical column, we consider the RF position obtained from the multi-unit activity of one recording session as representing the RF position of all the V1 single-units in the same recording session.

### Statistical analysis for firing rate modulation

For comparing the neuronal activity under passive and active viewing conditions, we compute two types of peri-stimulus time histograms (PSTH). The first type of the PSTHs is computed of the spiking activity at the beginning of every free-viewing trial representing the response to the onset of a stimulus image presented while the monkey was keeping a fixation at the center of a blank (except for the fixation spot) screen. We consider this condition as the passive viewing condition. The second type of the PSTHs is computed over the following free-viewing period, representing the response to parts of the stimulus image evoked by fixations following self-initiated saccades. We call this the active viewing condition. To compare the two, we align both to the beginning of the period when visual input is reaching the system, i.e., in the passive condition to the image onset (img-on), and in the active condition to the fixation onset (fix-on). An adaptive kernel width selection method^71^ is used to obtain smoothed versions of the PSTHs in continuous time at a temporal resolution of 2 ms.

We first tested if the firing rate at the peak of the PSTH was significantly higher than a baseline. For doing that we constructed, for every single-unit, a distribution of spike counts across img-ons or across fix-ons, depending on the PSTH type, by counting the spikes within a 20 ms wide bin that was centered around the peak time of the respective PSTH. The baseline is considered as the distribution of spike counts, sampled within exclusive 20 ms wide bins during the pre-trial or trial periods depending on the condition, as explained in the following. In the passive condition, the baseline count distribution was measured in the interval between − 1 s and 0 s from img-on. In the active condition (i.e., free-viewing) the counts are taken within the interval between 1 s after img-on and the end of the image presentation. This choice of the baseline for the active condition was taken because there is no “pre-stimulus” time interval during a free viewing trial; we decided to define the baseline as the overall spike count distribution during the whole free viewing period (except for the first 1 s, in order to exclude possible influences of img-on). The significance of firing rate peak was then tested by performing a Kolmogorov–Smirnov test (alpha = 0.05) on the peak spike count distribution and the baseline spike count distribution.

### Contrast and orientedness of fixated image

To study the influence of low-level visual features of the stimulus images on the spiking activity, we computed the contrast and orientedness of the image patch that fell into the receptive field of the recorded neurons. For every img-on and fix-on, a circular image patch located at the receptive field position relative to the current fixation point was cut out from the stimulus image. The size of the patch was set to be large enough to cover the whole receptive field. Then the image patch was converted to grayscale by computing the luminance at each pixel in the patch, the mean luminance was subtracted from all pixels, and the whole patch was multiplied by a two-dimensional gaussian kernel with the standard deviation of 0.25 times the diameter of the receptive field, such that the luminance values around the edge of the circular patch smoothly decay to zero, i.e., the mean luminance of the patch. The median diameter of the receptive fields from all recordings was 0.59° (Supplementary Fig. 2), which corresponds to 498.6 pixels contained within 2 standard deviations of the gaussian kernel. After this preprocessing, a two-dimensional Fourier transform was applied to the image patch to obtain the two-dimensional power spectrum ${P}_{{f}_{x},{f}_{y}}$ of the patch, where ${f}_{x}$ and ${f}_{y}$ indicate spatial frequencies in the horizontal and the vertical direction, respectively.

To quantify the contrast of the image patch, the power spectrum ${P}_{{f}_{x},{f}_{y}}$ was reduced to a one dimensional power spectrum ${P}_{f}={\sum }_{{f}_{x}^{2}+{f}_{y}^{2}={f}^{2}}{P}_{{f}_{x}, {f}_{y}}$, representing contrasts at different spatial frequencies *f*. To account for spatial frequency sensitivity of V1 neurons, a Gaussian kernel with the mean of 3.0 cycle/° and the full-width at half-maximum of 1.5 cycle/° according to De Valois et al.^72^ was multiplied to ${P}_{f}$, and then the power of all frequencies were summed up to yield a contrast measure.

To quantify the orientedness of the image patch, the power spectrum ${P}_{{f}_{x},{f}_{y}}$ was reduced to another one dimensional power spectrum ${P}_{\theta }={\sum }_{\arctan{\theta}={f}_{y}/{f}_{x}}{P}_{{f}_{x},{f}_{y}}$, representing contrasts of edges in different orientations. Then the orientedness of the patch is quantified as ${\sum }_{-\pi \le \theta <\pi }{P}_{\theta }{e}^{i\theta }/{\sum }_{-\pi \le \theta <\pi }{P}_{\theta }$.

### Sparseness

For every single-unit, we also computed the sparseness of the spiking activity in response to img-ons or fix-ons, separately. The activity in response to a single img-on or fix-on was quantified by the number of spikes emitted during a 100 ms time window centered at the time of the peak of the corresponding PSTH. Given the spike counts for all img-ons or fix-ons for a single-unit, we computed the lifetime sparseness *S* of the response spiking activity of this unit separately for the active and the passive condition using the following formula﻿^18,27^:

1 $$S=\frac{1-a}{1-\frac{1}{N}},$$

2 $$a=\frac{{\left({\sum }_{i=1}^{N}\frac{{r}_{i}}{N}\right)}^{2}}{{\sum }_{i=1}^{N}\frac{{r}_{i}^{2}}{N}},$$

where ${r}_{i}$ is the spike count for the *i-*th img-on or fix-on and *N* is the total number of img-ons or fix-ons. The sparseness *S* takes a value between 0 (when the unit responded equally to all img-ons or fix-ons, thus is least sparse) and 1 (when the unit responded to only one img-on or fix-on, thus is maximally sparse). Note that the sparseness is related to but not determined solely by stimulus preference/selectivity of the neuron. What we intend to quantify here with this sparseness measure is the variability of the responses of a single-unit to individual img-ons or fix-ons.

Given the sparseness of all single-units for img-on responses or fix-on responses, we constructed, separately for each unit class, two distributions of sparseness values: one from the img-on response sparseness, representing the sparseness in the passive viewing condition, and the other from the fix-on response sparseness, representing the sparseness in the active viewing condition. The difference in sparseness in the two conditions was tested for significance by performing a Wilcoxon signed-rank test (alpha = 0.05) on the two respective sparseness distributions.

#### Statement of accordance with arrive guidelines

The reporting in this study follows the recommendations in the ARRIVE guidelines.

## Supplementary Information

Supplementary Figures.

## Data Availability

All data and analysis codes are available from the corresponding author upon request.
