# Supplementary material for: Latency shortening with enhanced sparseness and responsiveness in V1 during active visual sensing
Source: Sci Rep. 2022 Apr 11;12:6021. doi: 10.1038/s41598-022-09405-4 (PMC9001710; doi:10.1038/s41598-022-09405-4)
Supplement: Supplementary file 1 — Supplementary Figures. [file 41598_2022_9405_MOESM1_ESM.docx]

# SUPPLEMENTARY INFORMATION


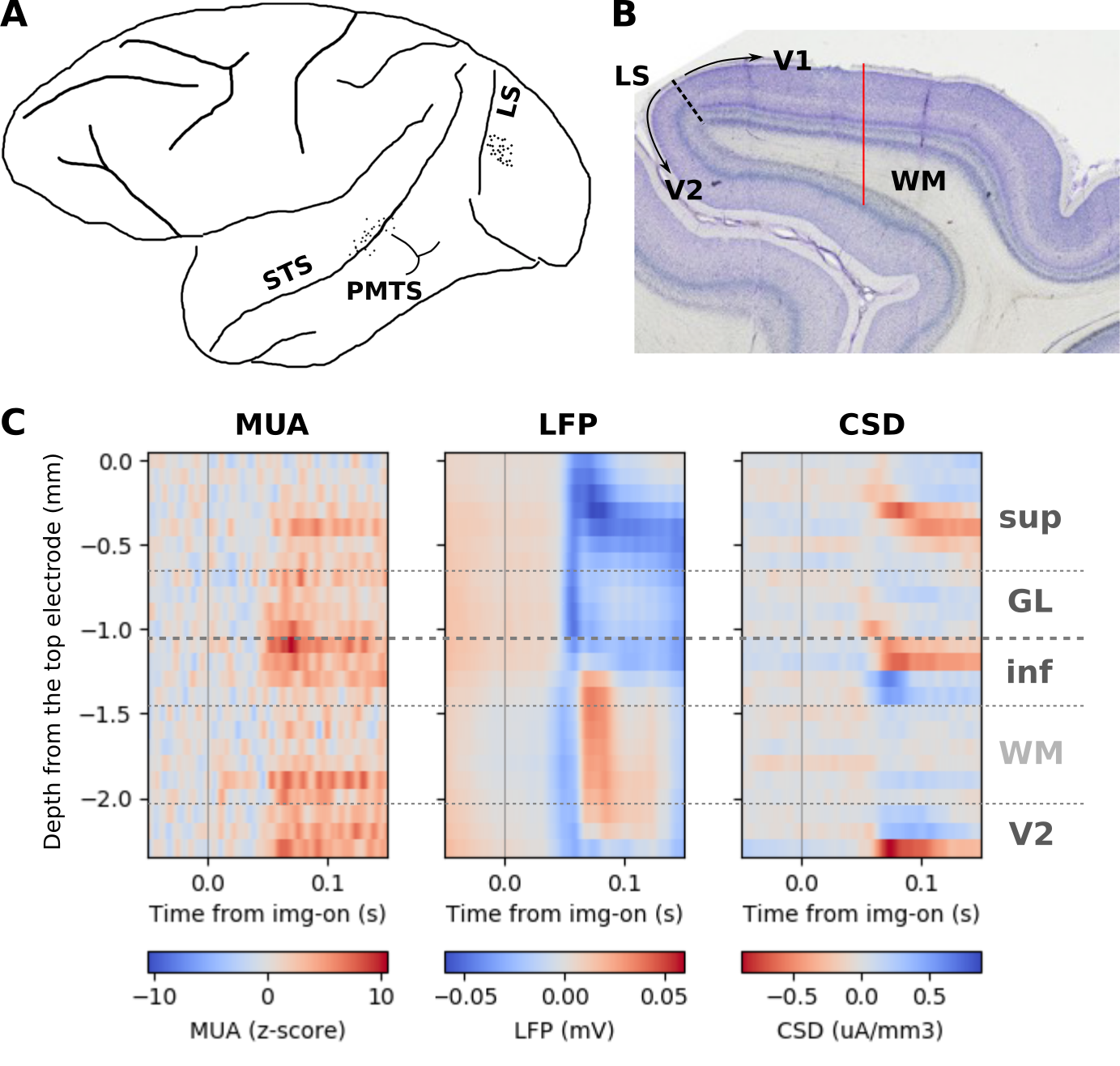


**Supplementary Figure 1.** Laminar recording in V1. (A) Electrode insertion sites in V1 and IT of monkey 2. Dots indicate the positions where the electrodes were inserted. LS: lunate sulcus; STS: superior temporal sulcus; PMTS: posterior middle temporal sulcus. (B) The brain section contains one of the electrode insertion positions in V1. The red line indicates the estimated electrode penetration track. In this experiment the electrode tip penetrated through the white matter and reached V2. LS: lunate sulcus; WM: white matter. (C) Laminar profile of neuronal activity in response to image onset. The data were recorded in the same experiment as shown in B. Multiunit activity (MUA; left) and local field potential (LFP; center) show distinct response activity. The current source density (CSD; right) estimated from the LFP shows a characteristic pattern of sink(red)-source(blue) pair, which marks the boundary between layer 4c and layer 5 (thick dashed line). The depth relative to this boundary defines the other layer boundaries (thin dashed lines; see Methods for details). Sup: supragranular layer; GL: granular layer; inf: infragranular layer; WM: white matter.


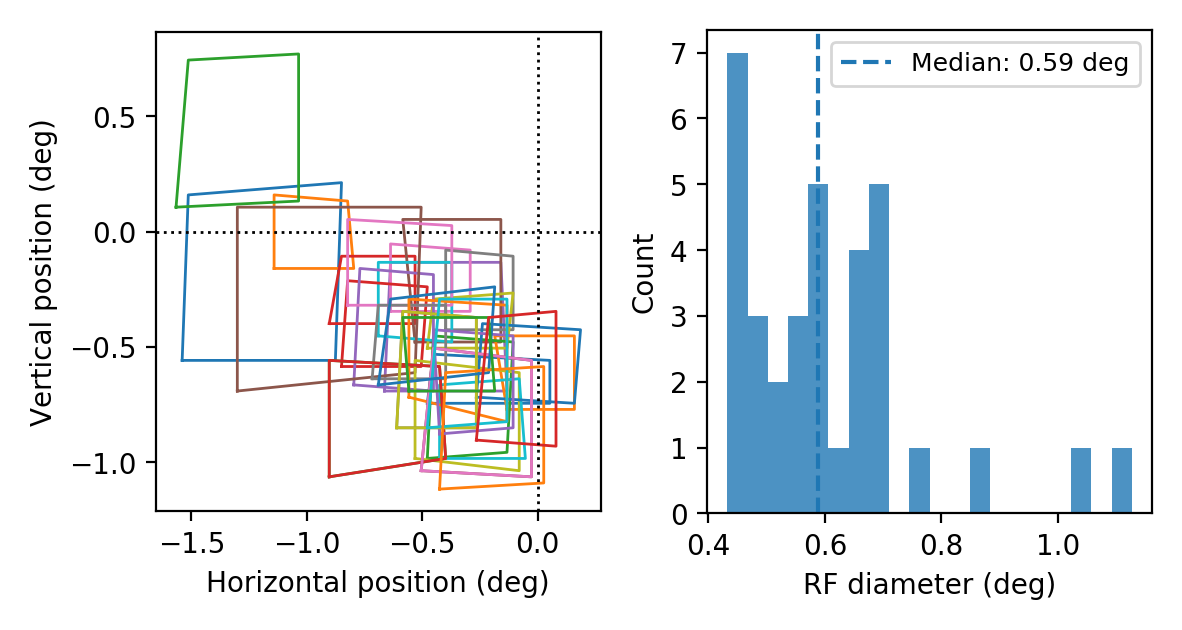


**Supplementary Figure 2.** Receptive field (RF) positions and sizes. (Left) Positions of the RFs from all recordings. Quadrangles of different colors represent the positions of RFs from different recordings. (Right) Histogram of the RF sizes in diameter. The median RF diameter was 0.59 deg.
